# Detection of lymph node metastasis in lung cancer patients using a one-step nucleic acid amplification assay: a single-centre prospective study

**DOI:** 10.1186/s12967-019-1974-4

**Published:** 2019-07-22

**Authors:** María Escalante Pérez, María Teresa Hermida Romero, Begoña Otero Alén, Mónica Álvarez Martínez, Ricardo Fernández Prado, Mercedes de la Torre Bravos, Ángel Concha López

**Affiliations:** 1 Biobank of A Coruña, INIBIC, A Coruña, Spain; 2 Molecular Biology Area, Department of Anatomical Pathology, University Hospital Complex A Coruña, As Xubias 84, 15006 A Coruña, Spain; 3 Department of Anatomical Pathology, University Hospital Complex A Coruña, As Xubias 84, 15006 A Coruña, Spain; 4 Department of Thoracic Surgery, University Hospital Complex A Coruña, A Coruña, Spain

**Keywords:** Lung cancer, OSNA assay, Metastasis, Intraoperative, LN status, Keratin 19

## Abstract

**Background:**

The use of one-step nucleic acid amplification (OSNA) allows for lymph node (LN) metastasis to be detected rapidly and accurately. We conducted a prospective single-centre clinical trial to evaluate OSNA assay in detecting LN metastasis of lung cancer.

**Patients and methods:**

A total of 705 LNs from 160 patients with clinical stage IA to IVA lung cancer were included in this study. The LNs were divided and submitted to routine histological diagnosis and OSNA assay and the results were compared. We also examined keratin 19 expression of different histological types lung primary tumours.

**Results:**

When the cut-off value was set to 250 copies/µl, the concordance rate between the two methods was 96.17% and the sensitivity 97.14%. Discordant results were observed in 27 LNs of 21 patients. Most of these discordant results were molecular micrometastasis expressing a very low number of copies with negative histology. Most thoracic tumours were positive for keratin 19.

**Conclusions:**

Our data show that the OSNA assay might be a useful and sensitive method to diagnose LN metastasis in lung cancer and could be applied to intraoperative decision-making in personalised lung cancer surgery based on LN status and a more accurate staging of patients.

## Background

Lung cancer is one of the most frequent human cancers and the leading cause of cancer deaths in the world. In the clinical management of the disease, identifying the histological classification by stages is essential for an accurate therapeutic approach for the patients [1, 2]. Diagnostic methods, such as endobronchial (EBUS) and oesophageal ultrasounds (EUS), fine-needle aspirations, or mediastinoscopies, are carried out to take samples of mediastinal lymph nodes (LNs) to perform the pre-surgical staging of the patient [3]. In patients with an early pathological stage, once the main tumour has been resected, the regional LNs are studied for the pathological staging that will define the treatment [2]. Traditionally, this step has been performed through microscopic examination of histological sections, the sensitivity of which can be increased by immunohistochemistry (IHC). Currently, the molecular detection of keratin 19 (KRT19) is a routine method in the clinical practice of breast cancer [4]. KRT19 is a type I keratin with a low molecular weight. It is part of the cytoskeleton of numerous epithelial cells of different organs, but is not detected in any of the cellular components of the LNs. Furthermore, it is highly expressed in epithelial tumours, so it is an appropriate biomarker for detecting micro- and macrometastasis. The one-step nucleic acid amplification (OSNA) assay is an automated molecular diagnostic assay that analyses the entire LN tissue. The reaction is based on rapid nucleic acid amplification technology, a loop-mediated isothermal amplification (RT-LAMP), to quantify KRT19 mRNA expression [5, 6]. The expression rate of KRT19 mRNA correlates with the size of the metastatic foci. In the last few years, the OSNA assay has been developed as an alternative intraoperative method for detecting of tumour metastasis in breast, gastric, and colorectal cancers [4, 7, 8]. Additionally, the feasibility of OSNA for other malignancies like thyroid, endometrial, head and neck and bronchopulmonary carcinomas is being investigated [9–12]. Only a few reports have been published about the potential of the OSNA assay in non-small cell lung cancer (NSCLC) patients [10, 13–15]. In this study we examined 705 LNs of 160 patients with lung cancer by OSNA assay. It represents the biggest cohort of patients and single measured LNs and one of only two reports outside of Japan. Furthermore, this is a single-centre study, with the subsequent advantage of a strictly standardised protocols and methodology. All LNs were divided to be evaluated by routine histological diagnosis or OSNA assay and the results were compared. We also evaluated the KRT19 expression rate in different types of primary thoracic tumours by IHC and OSNA. The purpose of the project was to test whether the OSNA assay is an accurate, feasible and adequate method for analysis of LN metastasis in lung cancer patients both in intraoperative and definitive pathological diagnoses.

## Patients and methods

### Patients and human samples

This study was conducted as a single-centre clinical performance study between July 2015 and December 2018. The study was done in compliance with the Declaration of Helsinki. Approval from the Clinical Research Ethics Committee, patients written informed consents custody and sample storage was managed by the Biobank of A Coruña.

A total of 705 LNs from 160 patients with lung cancer and a clinical stage IA to IVA according to the latest WHO classification [16] who underwent pulmonary resection by video-assisted thoracoscopic surgery (VATS) at University Hospital Complex of A Coruña, were included in this study. Patients with prior neo-adjuvant chemotherapy or radiotherapy, recurrent disease, or any previous or synchronous malignancy were excluded from this study. Of the included patients, 15 of them presented intestinal adenomas with low grade dysplasia; and 7 patients had suffered non-infiltrating carcinomas of different types within the 5 years before the start of the study. Furthermore, 9 additional patients with diverse granulomatous diseases were included as negative controls: 5 sarcoidosis, 3 tuberculosis and 1 aspergillosis. Demographic parameters and baseline characteristics of the patients were recorded and are shown in Table 1.

**Table 1:** Baseline characteristics of the patients

Variables	No.	%	Variables	No.	%
Patients	160		Pathological stage	160	
Male	115	71.88	IA1	5	3.13
Female	45	28.13	IA2	25	15.63
Age (year)			IA3	20	12.50
Mean (range)	65.07 (22–85)		IB	31	19.38
Surgical type of resection	160		IIA	11	6.88
Bi-lob	7	4.38	IIB	39	24.38
LID	22	13.75	IIIA	24	15.00
LII	18	11.25	IIIB	4	2.50
LSD	63	39.38	IVA	1	0.63
LSI	46	28.75	Dissected lymph nodes	705	
BPI	1	0.63	Hilar	72	10.21
LMD	3	1.88	Interlobar	52	7.38
Histology	160		Paratraqueal	184	26.10
Adenocarcinoma	94	58.75	Pulmonary ligament	38	5.39
Squamous cell carcinoma	37	23.13	Peribronchial	39	5.53
Adenosquamous carcinoma	5	3.13	Subcarinal	225	31.91
Small cell lung carcinoma	6	3.75	AP window	75	10.64
Large-cell carcinoma	7	4.38	Other	20	2.84
Carcinoid	8	5.00	Pathological N status	160	
Lymphoepithelioma like carcinoma	1	0.63	pN0	118	73.75
Carcinoma NOS	1	0.63	pN1	25	15.63
Pleomorphic carcinoma	1	0.63	pN2	17	10.63

### Preparation of LNs

**Fig. 1:**
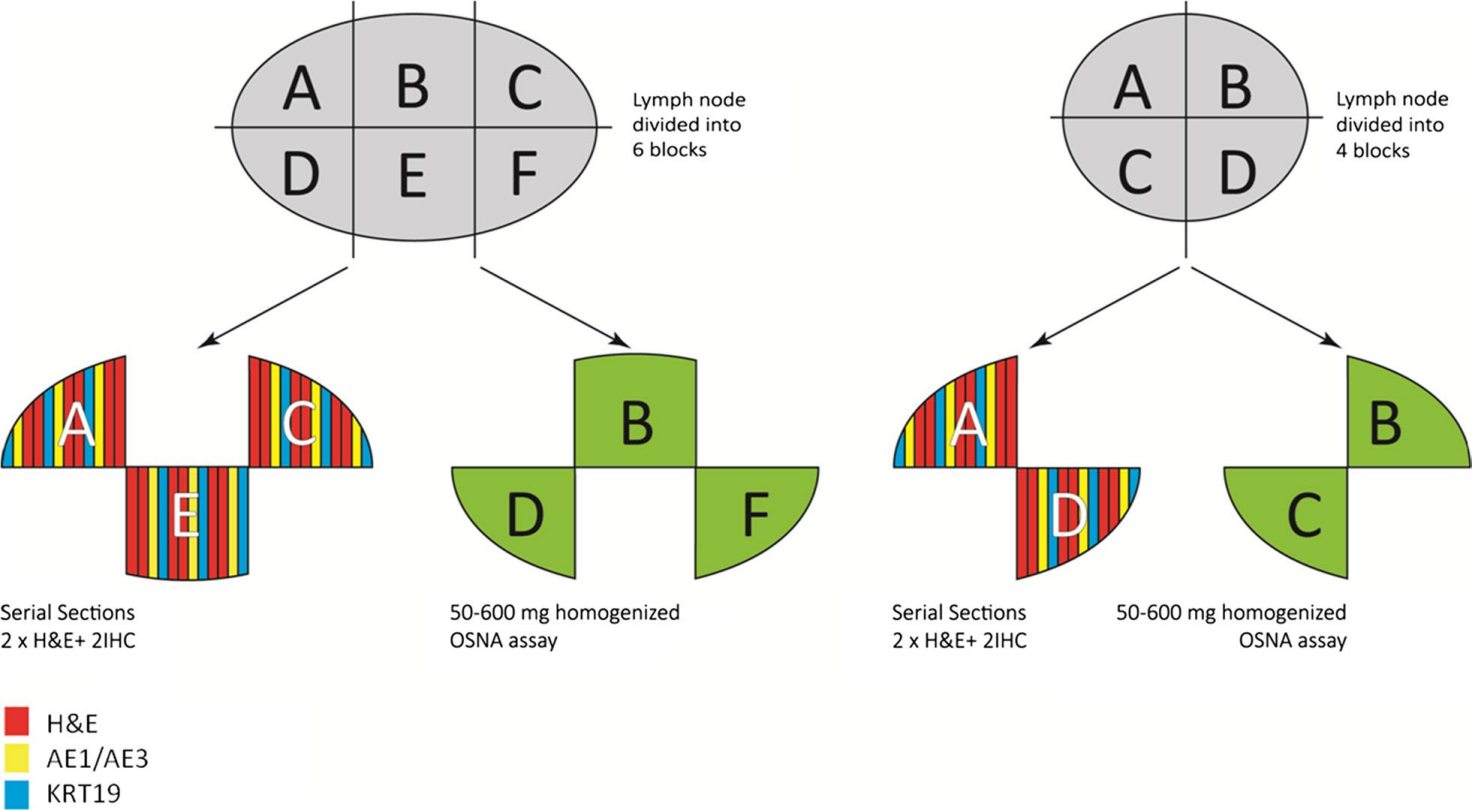
Preparation of a lymph node for OSNA assay and histological examination. Non-adjacent blocks were alternatively used for either the histological examination or the OSNA assay. For histopathological study, serial sections from the same tissue blocks were analysed by H-E and IHC. The two first sections was stained with H-E and the subsequent two using AE1/AE3 and KRT19 mouse monoclonal antibodies. The remaining resected lymph node was analysed by OSNA assay in single homogenized from 50 to 600 mg

After removal, LNs with a minimal weight of 0.1 g and a maximal of 1.2 g were immediately divided into 4 or 6 blocks depending on their size (4 blocks ≤ 1 cm; 6 blocks ˃ 1 cm). Non-adjacent blocks were alternatively used for either the histological examination or the OSNA assay as shown in Fig. 1.

### Histological examination

The haematoxylin/eosin (H/E)-stained histological slides from formalin-fixed, paraffin-embedded (FFPE) of LN surgical specimens and dissection were examined by two expert pathologists (THR, AC) blinded to the OSNA results. The pathological staging and histological diagnosis were based on the latest AJCC Cancer Staging Manual of the TNM classification for lung cancer [17], and the WHO Classification of Tumours of the Lung, Pleura, Thymus and Heart [16], respectively, with the aid of immunohistochemical panels. Any discordant results were resolved by consensus.

### OSNA assay

We analysed 705 LNs and 40 primary tumours from 160 patients and 9 granulomatous diseases with the OSNA assay. We used KRT19 mRNA as a marker and the protocol was as previously described [4]. In summary, the resected LNs or primary tumours were homogenised using Lynorhag lysis buffer (Sysmex Corp., Hyogo, Japan). The KRT19 mRNA in each lysate was amplified using a Lynoamp BC gene amplification reagent (Sysmex Corp., Hyogo, Japan), then a 20-μl sample of each lysate was subjected to a RT-LAMP reaction. Amplification of KRT19 mRNA was detected by measuring the rise time based on a standard curve using an RD-100i (Sysmex Corp., Hyogo, Japan). The results of the assay were expressed as KRT19 mRNA copy numbers per microliter. The cut-off for micrometastasis was set at 250–5000 copies/µl and over 5000 copies/µl was considered macrometastasis; less than 250 copies/µl was considered negative.

### Immunohistochemical analysis

When discordance results were observed between the OSNA assay and common pathological examination, serial sections from the same tissue blocks were analysed by IHC. Specimens were cut into blocks 3.5 µm thick. The first section was stained using a mouse monoclonal antibody cocktail reacting with human cytokeratins AE1/AE3 PCK26 (Anti-Pan Keratin, Ventana Medical Systems, TX, USA). The second section was stained using a mouse monoclonal antibody anti-KRT19 (clone A53-B/A2.26, Ventana Medical Systems, TX, USA). An immunohistochemistry was performed according to previous protocols using the BenchMark Ventana immunostainer automated method (Ventana Medical Systems, TX, USA). The procedure was as follows: deparaffinisation and rehydration using xylene and concentrated ethylene; heat-induced antigen retrieval; incubation with the primary antibodies; incubation with horseradish peroxidase-conjugated polymers (ultraView Universal DAB Detection Kit, Ventana Medical Systems, TX, USA); chromogenic reaction using DAB-H_2_O_2_; counterstaining with haematoxylin. Appropriate negative and positive controls were run parallel to the staining procedures. In addition, 160 lung resected primary tumours and 9 granulomatous diseases were also examined for KRT19 IHC expression, one representative sample from each patient.

Expression levels were classified based on an adapted Allred scoring system for quantification of hormonal receptors [18], and ERBB2 testing in breast cancer [19]. It is a semiquantitative system that takes into consideration the proportion of positive cells (scored on a scale of 0–5) and staining intensity (scored on a scale of 0–3). The proportion and intensity were then added to produce a total score range of 0 to 8. A score of 0–4 was considered negative, while a score from 5 to 8 was considered positive. Photographs were taken using an Olympus BX50 microscope and DP70 software (Olympus Corporation, Tokyo, Japan). Two different observers performed the evaluations independently. Any discordant results were resolved by consensus.

### Statistical analysis

Descriptive statistics have been used for characterising the clinical and pathological data of the patients in the study. The sample size has been determined mainly based on feasibility. The comparative analysis of the OSNA assay to the histological system was analysed using the χ^2^ test model. A unilateral significance level of 0.05% and a power of 90% was used to declare a result as statistically significant. The accuracy of the OSNA molecular method with respect to anatomopathological results was analysed by describing the proportion of non-inferiority, the kappa statistic and their respective confidence intervals (CI) of 95%.

## Results

### Correlation between the OSNA assay and the histological examination

A total of 705 LNs obtained from 160 patients with lung cancer in different stages were used for concordance analysis with the OSNA assay and the routine histological examination for staging. Of the 705 LNs, 644 were diagnosed by OSNA assay as negative, 34 with −micrometastasis and 26 with −macrometastasis (Tables 2, 3). Two samples with isolated tumour cells (ITCs) were found in our study, intrasinusoidal and in intravascular thromby in lymphatic channel. Both positive by H-E. We compared the validity (sensitivity and specificity) and reliability (positive (PPV) and negative predictive value (NPV)) of the OSNA method versus the gold standard (pathological study: histology and IHC). Accepting only macro-macrometastasis as positive, as established in breast cancer, our results showed a 99.29% concordance rate with a kappa statistic of 0.894 (95% CI 80.20–98.60%). The sensitivity of the OSNA assay as compared to the pathological examination was 95.65% (95% CI 79.01–99.78%) and specificity was 99.41% (95% CI 98.50–99.77%). PPV and NPV was 84.62% and 99.85%, respectively.

**Table 2:** LNs results

OSNA		H-E/IHQ	
		Positive	Negative
++	Positive	22	4
	Negative	1	678
++/+	Positive	34	26
	Negative	1	644

+: micrometastasis; ++: macrometastasis

**Table 3:** OSNA

	++	+ and ++
Concordance (%)	99.29	96.17
Specificity (%)	99.41	96.12
Sensitivity (%)	95.65	97.14
PPV (%)	84.62	56.67
NPV (%)	99.85	99.84

When the cut-off value was set as 250 copies/μl, as reported in previous studies on other organs [4], we obtained a 96.17% accuracy with a kappa statistic 0.697 (95% CI 59.00–80.40%), 97.14% sensitivity, 96.12% specificity; and values of 56.67% PPV and 99.84% NPV (Table 3). These results are similar to those obtained in other similar studies [12–15].

Our results showed that with both comparisons the p-value from the non-inferiority test between OSNA and gold standard was < 0.0001 with a 95% of CI, i.e. OSNA assay is a method non-inferior to gold standard in the diagnosis of LN metastasis.

### Analysis of discordant cases

We found a high correlation between anatomopathological diagnosis by H/E and the results obtained by OSNA assay, however discordant results were observed in 27 LNs (21 patients), less than 5% of all LNs analysed. Twenty-six lymph nodes were found negative for H/E and IHC and positive for OSNA. On the other hand, only one LN was negative by the OSNA assay and diagnosed as metastatic in the histological study. Twenty-two LNs were histologically negative but considered positive for −micrometastasis by the OSNA assay. Most of these cases presented several relatively low mRNA copies (less than 1000 copies). Two of these patients with discordant -micrometastasis (no. 15 and no. 16) also showed −macrometastasis levels of KRT19 in other different LNs by the OSNA assay and were also negative by the pathological examination. Curiously, one of them was diagnosed histologically metastatic in the subsequent pathological study of whole surgical specimen and staged as pN2 (Table 4).

**Table 4:** Details of the discordants LNs

Patient ID	Number of discordant LNs	OSNA assay (copies)	Histological examination	Metastasis in other LNs
6	2	410	Negative	Negative
		270	Negative	
8	1	1100	Negative	
12	1	720	Negative	Negative
14	1	270	Negative	Negative
15	3	1600	Negative	Positive
		94,000	Negative	
		31,000	Negative	
16	2	720	Negative	Negative
		170,000	Negative	
18	1	950	Negative	Negative
20	1	260	Negative	Negative
26	1	2200	Negative	Negative
30	1	410	Negative	Negative
39	1	1400	Negative	Positive
48	1	470	Negative	Negative
51	2	530	Negative	Negative
		360	Negative	
61	1	1100	Negative	Positive
66	1	940	Negative	Negative
67	1	270	Negative	Negative
74	1	410	Negative	Positive
84	1	5800	Negative	Negative
87	2	410	Negative	Negative
		3400	Negative	
115	1	830	Negative	Negative
94	1	–	Positive	Negative

**Fig. 2:**
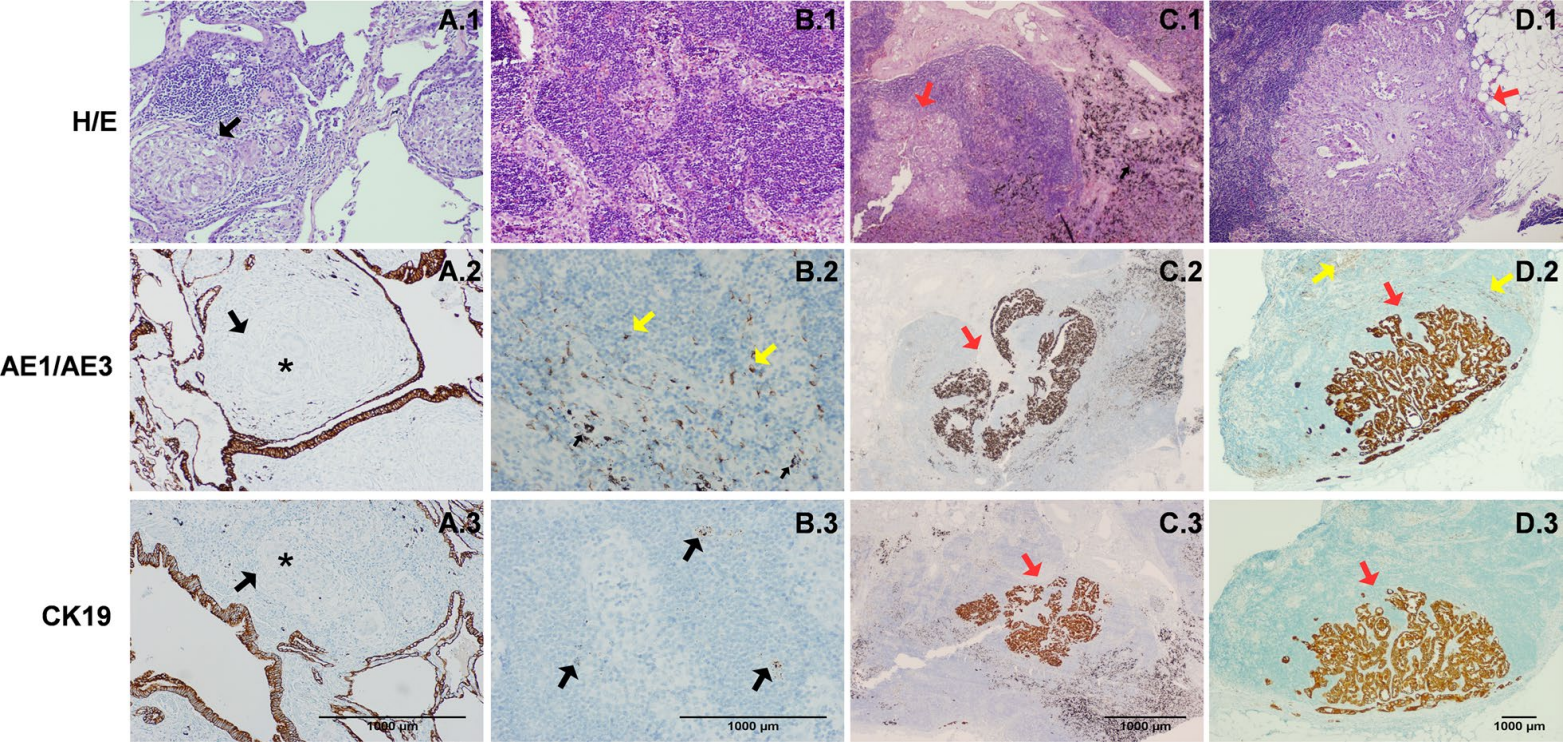
Haematoxylin/eosin, pan-keratin AE1/AE3 and KRT19 expression in sarcoidosis and lymph node samples. **a** Sarcoidosis. The epithelial cells (asterisk) of the granulomas (black arrows) are negative for both AE1/AE3 and KRT19 (×10). **b** Non-metastatic lymph node. The lymph nodes have isolated cells that express AE1/AE3 (accessory cells; yellow arrows), but not KRT19. Anthracotic pigment (black arrows) (×10). **c** Micrometastatic lymph node. Lymph node with a deposit of 1.8 mm of positive neoplastic cells for AE1/AE3 and KRT19 (red arrows) (×4). **d** Macrometastatic lymph node. Lymph node with expression of AE1/AE3 in accessory cells (yellow arrows) and AE1/AE3 and KRT19 in the tumour cells (macrometastasis of 6 mm; red arrows) (×2)

Of the remaining 4 discordant LNs showing −macrometastasis by the OSNA assay, in three of them we detected very high levels of mRNA copies. However, in all the cases no metastatic deposits were found by conventional methods or IHC for KRT19 and AE1/AE3 to ensure the non-existence of isolated neoplastic cells. Although LNs had some non-epithelial cells expressing AE1/AE3, no KRT19 expression was observed in those cells (Fig. 2).

The only negative LN by OSNA and positive by H/E presented a metastatic intranodal focus of 4 × 5 mm in size. The size of the −macrometastasis suggested that this discordance could have been due to a sampling bias.

### KRT19 expression levels in primary lesions, correlation between OSNA assay and IHC

**Fig. 3:**
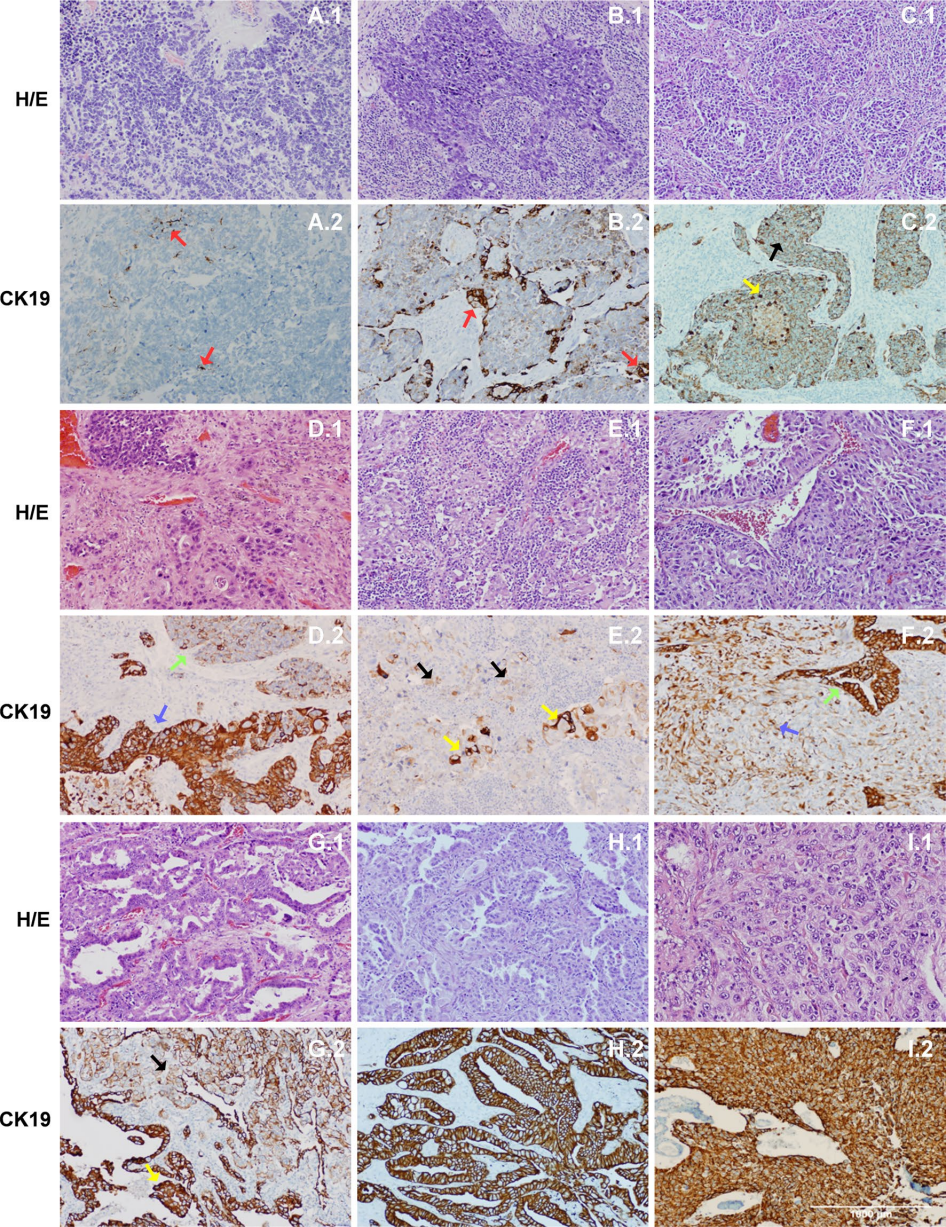
Haematoxylin/eosin and KRT19 expression in lung primary tumours. **a** Small cell carcinoma. Only isolated cells are positive for KRT19 (˂1%; red arrows). **b** Large cell carcinoma. Heterogeneous immunostaining with a positive pattern of ˃ 10% of tumour cells (red arrows). **c** Large cell carcinoma. Heterogeneous expression of numerous neoplastic cells with different levels of expression (black and yellow arrows), although obvious in all of them. **d** Mixed neuroendocrine carcinoma. Differential expression between the two components of the neoplasm: weak and patchy in the small cell (green arrow) and intense and diffuse in the large cell component (blue arrow). **e** Lymphoepithelioma-like carcinoma. Neoplastic cells show a faint and heterogeneous pattern (black arrows) although some elements show high expression of KRT19 (yellow arrows). **f** Pleomorphic carcinoma. Intense immunostaining in the glandular component (green arrow) and heterogeneous pattern of less intensity in the sarcomatoid fraction (blue arrow). **g** Adenocarcinoma. Heterogeneous but obvious pattern of expression of KRT19 (black and yellow arrows). **h** Adenocarcinoma. Intense and diffuse positivity for KRT19 in the whole tumour. **i** Squamous cell carcinoma. High expression of KRT19 in all tumour cells. Objective magnification ×10

We examined KRT19 expression by IHC in all primary tumours of the study and 9 samples of patients with granulomatous diseases. In addition, 40 of these 160 primary tumours and the 9 granulomatous disease samples were examined also by OSNA assay to study the correlation between both diagnostic methods. The expression levels by IHC were quantified by the adapted Allred method scoring system, as previously mentioned. All the lesions of granulomatosis were negative for KRT19 by OSNA and IHC. Regarding primary tumours, we have analysed 19 adenocarcinomas, 13 squamous-cell carcinomas, 2 adeno-squamous carcinomas, 2 large-cell carcinomas, 1 mixed neuroendocrine tumour, 1 lymphoepithelioma-like carcinoma and 2 small-cell carcinomas (Table 5). All tumours were positive by the OSNA assay apart from one small-cell carcinoma. Furthermore, most of them showed high expression levels of KRT19 mRNA except one adenocarcinoma and one epidermoid carcinoma. The expression rate of all histological types apart from small-cell carcinomas was 100% by IHC and the OSNA assay. The protein expression detected for adenocarcinomas and adeno-squamous carcinomas was the maximum (median score (ms):8); and for the squamous carcinomas it was 7.9. Mixed neuroendocrine tumours and lymphoepithelioma-like carcinomas showed a score of 6 (both: 3 of positive cells + 3 of staining intensity) by the Allred system. Large-cell carcinomas showed a ms of 5.5 by IHC (more than 33% of cell expression and high intensity) (Fig. 3).

**Table 5:** KRT19 expression levels in the primary tumor

Histological type	Total	KRT19 expression OSNA			KRT19 IHQ		Expression rate	
	40	++	+	−	+	Median score	IHC (%)	OSNA (%)
Adenocarcinoma	19	17	2	0	19	8	100	100
Squamous carcinoma	13	12	1	0	13	7.9	100	100
Adenosquamous carcinoma	2	2	0	0	2	8	100	100
Large cell carcinoma	2	2	0	0	2	5.5	100	100
Mixed neuroendocrine tumor	1	1	0	0	1	6	100	100
Lymphoepithelioma-like carcinoma	1	1	0	0	1	6	100	100
Small cell carcinoma	2	1	0	1	1	5	50	50

We also examined 2 small-cell carcinomas. Only one was negative for KRT19 by both expression methods (ms of 4; 1 + 3, less than 1% of cell expression and high intensity). By the OSNA assay the positive primary tumour expressed high levels of KRT19, while the median score by IHC was 5.

## Discussion

Lung cancer is a disease with a high mortality. Some of the complexity associated with the pathology is due to its heterogeneous nature. Large-scale sequencing studies have revealed the complex genomic landscape of NSCLC and strong differences between lung adenocarcinomas and lung squamous-cell carcinomas [20]. However, both tumour types have an over 96% expression of KRT19, a commonly used marker for epithelial tumours [20]. In most pleural and lung tumours, positive rates for expression of KRT19 range from 70 to 100%. In small-cell carcinomas the expression rate is almost 60% [21–23]. But, the criteria for immunohistochemical scores are not protocolled. Literature shows different evaluation ranges, and the fact is that the positive threshold is not yet properly established for KRT19 in lung cancer. Nevertheless, this biomarker is widely expressed in bronchopulmonary malignancies. Moreover, all LN metastasis from KRT19-positive primary lung tumours express the protein by IHC. Surprisingly, deposits from KRT19 negative primary neoplasms can be positive for KRT19 in LNs too [20]. This fact is commonly observed in breast cancer, where 67% of KRT19 negative primary tumours show KRT19 positivity in LNs metastasis [24]. Our results in primary lung tumours showed high KRT19 expression by OSNA and IHC, not only in number of positive cells, but also in staining intensity. Only neuroendocrine carcinomas presented lower rates. These data suggest that KRT19 is an accurate biomarker for detecting metastasis in lung malignancies and that, unlike in breast cancer, performing IHC for KRT19 from the primary tumour site prior to OSNA analysis might not be necessary. In contrast, our samples of different granulomatous diseases were negative for KRT19 both by OSNA and pathological examination.

In this context, our present findings in 160 patients demonstrated that the OSNA assay successfully predicts LN metastasis in lung cancer, showing high specificity, concordant rates and NPV by comparison with the gold standard technique. These results are in accordance with the few previous studies in NSCLC that are similar [10, 13–15]. Furthermore, our research includes a larger number of patients and LNs analysed individually in a single-centre study. This fact implies a strictly standardised and normalised protocol for the performance of the whole process with an experienced surgical and diagnostic team in these procedures. However, this premise may have its weaknesses and limitations, so for the next step for validation of the technique in lung cancer, it would be necessary to conduct a multi-centre study in which not all variables and characteristics are controlled, in order to established our results.

Despite our high concordance rate, 27 LNs showed discordant results in 21 patients. Regarding −macrometastasis, 4 LNs were OSNA positive and pathological examination was negative, with two of them belonging to the same patient who underwent an N2 stage with other affected LNs. This case could be due to a sampling bias, as it was the only OSNA negative discordance with a positive histological diagnostic. LN metastasis are usually diagnosed with a postoperative pathological examination of permanent sections. However, with this method, small areas of metastasis between slices may go undetected, as may micro-micrometastasis or ITCs. Single tumour cells or small aggregates of tumour cells can be overlooked among the lymphocytes and sinusoidal histiocytes that could lead to a false-negative assessment [25]. Moreover, only a small part of the node is analysed each time. The great advantage of the OSNA assay is its ability to examine the whole LN as compared with the pathological examination, which scans only several sections. Furthermore, histological examination procedures for LNs are not standardised, and the inter-observer reproducibility of measuring metastatic tumour volume can occur [26]. This could lead to an under-stage pathology and the subsequent inappropriate treatment. Although KRT19 is present in healthy epithelial cells [10], the protocol for LN resection and processing in the laboratory prior to OSNA analysis avoids any potential false positives due to contamination. Likewise, the presence of benign epithelial inclusions is exceptional in mediastinal LNs, as well as its likelihood to cause a false-positive result. The differences and similarities between histopathological procedures and OSNA assay is shown in comparison Table 6.

**Table 6:** Technical comparison between histological procedures and OSNA assay

	Histological examination	OSNA assay
Tumour topography	Exhaustive study of tissue topography and detection of extracapsular extension	The assay do not enable the study of tumour topography, but adjacent lymph node tissue is analysed by complementary histological procedures
Morphological study	Study of metastatic deposits and isolated neoplastic cells. Minimisation of false positives like benignant epithelial inclusions (exceptional in mediastinal lymph nodes)	The assay do not enable the study of cellular and histological lymph node morphology
Sensibility	Lower limit of detection	More accuracy than histological procedures. Detection of occult micrometastasis
Whole lymph node analysis	Risk of sample bias. Slow and laborious study of whole lymph node	Fast, feasible and accurate whole lymph node analysis. Minimisation of false negatives
KRT19 quantification	Relative quantification of KRT19 expression by digital pathology	Absolute quantification of KRT19 expression that correlates with metastatic foci. The assay also allows the possibility of detecting total tumour load
Assay rapidity/Workflow	Fast analysis of a single intraoperative slide (less than 15 min)	Fast analysis of the whole lymph node (less than 25 min)
Methodology	Standardised procedures. Immunohistochemical scores and histological examination not protocolled	Normalised and standardised protocols. Unbiased results
Cost-effectiveness	Higher cost of time, personnel and resources for diagnostic	Lower cost-effectiveness ratio
Remnant sample	Remnant samples are available for histological and molecular analysis	Remaining lymph node lysates are available for subsequent molecular analysis

The high sensitivity of the OSNA assay also enables detecting of occult −micrometastasis missed by pathological examinations. We detected 22 −micrometastasis with a negative KRT19-IHC. The significance of these findings is not clear, nor are the implications in treatment decisions. Patients with pathological pN0 or pN1 diseases have heterogeneous outcomes [27–29]. Maybe these occult −micrometastasis are the key to explaining why some of these cases are classified as pN0 or pN1 after surgery, by means of conventional methods, progressing with worse clinical course. In breast cancer there are discrepancies about the presence of these microdeposits and whether they have do or do not have an impact on disease-free survival (DFS) and overall survival (OS) [30–32]. The same conflicting results are shown for lung cancer. Rena et al. [33] showed that LN ITCs and micrometastasis in pathological stage I NSCLC do not affect long term DFS. In contrast, other groups concluded that patients with ITCs or micrometastasis in LNs had higher recurrence rates and worse survival rates in early-stage NSCLC [34, 35]. Therefore, the cut-off point of the KRT19 copy number which is pathologically significant is critical. In this work we used the established parameters for breast cancer [4]. Do we need validation studies for the KRT19 mRNA copy numbers set up as cut-off for negative micro- and −macrometastasis in lung cancer? Or are breast cancer approximations accurate enough values applied to mediastinal LNs? The cut-off level of the OSNA assay used in previous studies in gastric, colorectal and lung cancer was also established at 250 copies/μl, considering that the KRT19 mRNA expression level is similar among the various types of malignancies. However, in head and neck cancer, new cut-off levels of 131 and 300 copies/μl were proposed by different studies [9, 36]. Head and neck tumours are squamous cell carcinomas and expression of KRT19 is a poor prognostic marker [37]. Most breast, gastric, colorectal, and between the 40 and 60% of lung malignancies, are adenocarcinomas. However, 20–30% are epidermoids and, thus, the cut-off point established for lung cancer could depend on the histological type of the tumour. For all these reasons, the limits of detection of the assay in bronchopulmonary malignancies must be established, monitoring the progression of patients with conflicting results, micrometastasis deposits and presence of ITCs. Furthermore, in lung carcinomas with low or no expression of KRT19, such as small cell carcinomas, the inclusion of a second or third marker could minimise false negatives and improve the specificity of the test.

On the other hand, in colorectal cancer, the amount of tumour burdens or total tumour loads detected by OSNA were showed to be an accurate LN pathological staging with potential prognostic implications [38]. Moreover, studies show that OSNA positivity in stage II colon cancer patients who are pN0 by H&E is associated with classical high-risk factors and has a prognostic value, suggesting that OSNA could be detecting patients as under-staged by histological techniques [39]. The total tumour burden pooled by RT-LAMP has not yet been analysed in lung cancer, but nevertheless could be a promising approach for future studies.

## Conclusions

Our findings show that the OSNA assay is a fast, feasible and accurate technique. It is a methodology that improves cost-effectiveness, accuracy and management of patients with an adequate clinical practice in pre-staging and pathological diagnosis. Furthermore, it is able to detect macrometastatic foci and small metastatic lesions and ITCs that are not identified by conventional techniques. The clinical and prognostic significance of such findings would also need to be evaluated with prospective studies, including a sufficient number of patients and an adequate follow-up period. The analysis of the whole LN by the OSNA assay could overcome the limitations of sample bias and the histological examination. In addition, the remaining LN lysate has proved to be adequate for subsequent molecular analysis [40]. In conclusion, this work addresses the validity of the procedure as an adequate method for diagnosis of LN metastasis in lung cancer.

## Data Availability

The datasets analysed during the current study are available from the corresponding authors on reasonable request.

## Abbreviations

EBUS: endobronchial ultrasounds
EUS: oesophageal ultrasounds
LN: lymph node
IHC: immunohistochemistry
KRT19: keratin 19
OSNA: one-step nucleic acid amplification
RT-LAMP: loop-mediated isothermal amplification
NSCLC: non-small cell lung cancer
VATS: video-assisted thoracoscopic surgery
H/E: haematoxylin/eosin
FFPE: formalin-fixed, paraffin-embedded
CI: confidence interval
ITCs: isolated tumour cells
PPV: positive predictive value
NPV: negative predictive value
DFS: disease-free survival
OS: overall survival

## Consents for publication

An informed consent for participation in the study was obtained from all patients.
